# Family and partner interpersonal violence among American Indians/Alaska Natives

**DOI:** 10.1186/2197-1714-1-7

**Published:** 2014-03-20

**Authors:** Katherine J Sapra, Sarah M Jubinski, Mina F Tanaka, Robyn RM Gershon

**Affiliations:** 1 Department of Epidemiology, Mailman School of Public Health, Columbia University, 722 W. 168th St, New York, NY 10032 USA; 2 Department of Health Policy and Management, Mailman School of Public Health Columbia University, 722 W. 168th St, New York, NY 10032 USA; 3 University of Illinois College of Medicine at Rockford, 1601 Parkview Ave, Rockford, IL 61101 USA; 4 Department of Epidemiology and Biostatistics, School of Medicine, University of California, San Francisco, 3333 California Street, San Francisco, CA 94118 USA; 5 Philip R. Lee Institute for Health Policy Studies, School of Medicine, University of California, San Francisco, 3333 California Street, San Francisco, CA 94118 USA

**Keywords:** American Indians/Alaska Natives, Native Americans, Child abuse, Child neglect, Elder abuse, Domestic violence, Sexual assault, Violence against women, Rape

## Abstract

Family and partner interpersonal violence are common among American Indian/Alaska Native (AI/AN) populations. AI/AN women have the second highest prevalence of violence against women among all racial/ethnic groups in the United States, and child abuse prevalence rates in AI/AN populations are among the highest. Elder abuse in AI/AN is also an important concern, although data on this are sparse. This review describes the epidemiology of child abuse, violence against women, and elder abuse among AI/AN, including prevalence and associated risk factors. The authors discuss potential reasons for the high burden of interpersonal violence among AI/AN, including common risk factors. Important limitations in existing literature are also highlighted, along with recommendations for future research on this topic.

**Electronic supplementary material:**

The online version of this article (doi:10.1186/2197-1714-1-7) contains supplementary material, which is available to authorized users.

## Introduction

In comparison to other races/ethnicities, American Indians/Alaska Natives (AI/AN) have higher rates of interpersonal violence. Child abuse, violence against women (VAW), and elder abuse are important contributors to the increased risk of morbidity and mortality among AI/AN. In this article, we present an overview of the prevalence and risk factors for family and partner interpersonal violence in AI/AN living in the United States and discuss research gaps, with recommendations for future research directions.

The World Health Organization defines interpersonal violence as “the intentional use of physical force or power, threatened or actual, against another person that either results in or has a high likelihood of resulting in injury, death, psychological harm, maldevelopment or deprivation (World Health Organization 2002).” Interpersonal violence is subdivided into family/partner (related individuals) and community (unrelated individuals) violence. This paper focuses on the former, which includes child, intimate partner, and elder abuse (World Health Organization 2002), and also touches upon community violence of a sexual nature (e.g., rape by acquaintances and strangers).

Multiple small-scale studies and national surveys have demonstrated a higher prevalence of interpersonal violence among AI/AN populations compared to the general U.S. population. Child abuse and neglect and VAW are alarmingly high. Child abuse and neglect prevalence in AI/AN communities are among the highest of any racial/ethnic group, and VAW affects the majority of AI/AN women. Elder abuse is a concern for AI/AN tribal leaders, though it is not well-characterized.

As in other populations, obtaining accurate prevalence data can be challenging. While some population-based studies among AI/AN living on reservations have been conducted (Harwell et al. 2003; Manson et al. 2005), much of the existing literature on interpersonal violence among AI/AN relies on existing data sources, particularly records from law enforcement, governmental agencies, and health care services. Only those who report abuse to police or seek medical care for injuries are included, and victims may be hesitant to report crimes due to shame and fear of retaliation. Furthermore, overlapping and conflicting jurisdictions between tribal, state, federal authorities impede reporting of abuse and neglect (Cross and Simmons 2008). State reporting requirements typically do not extend to AI/AN living on tribal lands, and Fox (2003) estimates that only about 42-61% of AI/AN child abuse cases reach federal reporting systems. These obstacles are further complicated by inconsistent definitions of abuse and record-keeping for AI/AN, and a lack of resources for tribal workers responsible for reporting cases. Estimating the prevalence of interpersonal violence from existing data sources is made more difficult as tribal nations do not have jurisdiction over non-AI/AN (Deer 2004) and 70% of violent crime against AI/AN is perpetrated by non-AI/AN (Greenfeld and Smith 1999).

Valid prevalence estimates depend upon accurate numerators (number of cases) and denominators (population at risk). The challenges in reporting described above contribute to inaccurate prevalence estimates, as the numerator obtained from these sources will be artificially low. These methods of case ascertainment may also introduce selection bias as victims who reach the attention of service providers may have different risk factor profiles than those who do not. While these limitations are not unique to studies among AI/AN, they remain an impediment to our understanding of the burden of interpersonal violence in this population.

The studies reviewed in this article fall into either of two categories: cross-sectional surveys and ethnographic studies, generally with data collected fromretrospective self-reporting or review of records. Due to the absence of longitudinal studies, incidence rates of interpersonal violence in AI/AN cannot at this time be ascertained or compared between subpopulations.

It is believed that the historic maltreatment of AI/AN and the transmission of these historical injuries through generations contribute to the high prevalence of interpersonal violence among AI/AN (Brave Heart 1998). Early annihilation efforts by the U.S. government, including warfare and purposeful spread of infectious diseases (DeBruyn et al. 2001), were followed by assimilation efforts through placement of AI/AN children in boarding schools and urban relocation programs (Bell and Lim 2005; Brave Heart 1998). Assimilation efforts also may have led to loss of culture and traditions, resulting in increased prevalence of dysfunctional family life and mental disorders, which are additional risk factors for interpersonal violence (Brave Heart 1998; Duran et al. 2009; Duran and Duran 1995; Pavkov et al. 2010). In the case of boarding schools, traditional familial and parenting models were dissolved and replaced with institutional care, which often modeled negative behaviors, such as abuse and neglect.

Scholars have suggested that historical trauma contributes to interpersonal violence in AI/AN populations through several mechanisms. Brave Heart (2003) posits that many AI/AN turned to alcohol and other substances as a coping mechanism. Increased substance use may thus serve as a link between historical trauma and violence (Arbuckle et al. 1996; Berlin 1987; DeBruyn et al. 1992; Horejsi et al. 1992; King 1999; Luna-Firebaugh 2006; Nelson et al. 1996; Piasecki et al. 1989; Robin et al. 1998).

## Review

### Prevalence of child abuse

While no standard definition of child maltreatment is used by all reporting and researching entities (Earle and Cross 2001), under federal law, child abuse and neglect are defined as, “any recent act or failure to act on the part of a parent or caretaker, which results in death, serious physical or emotional harm, sexual abuse or exploitation, or an act or failure to act which presents an imminent risk of serious harm” (Child Abuse Prevention and Treatment Act 2013, p. 6). The per capita rate of substantiated report of child abuse and neglect for AI/AN children 14 years or younger is one for every 30 children, compared to one for every 58 in the general population (Fox 2003). Nationally, AI/AN children have higher rates of reported abuse and neglect than their white counterparts, but have similar or lower rates compared to African American children (U.S. Department of Health and Human Services 2010). However, one analysis of data from Department of Health and Human Services’ National Child Abuse and Neglect Data System (NCANDS) found lower rates of physical and sexual abuse in AI/AN children than in non-Hispanic White children (Earle and Cross 2001). This observation is in discrepancy with findings from other studies, but may be due to underreporting, as AI/AN abuse cases investigated by tribal law enforcement or the Federal Bureau of Investigations are not always reported to NCANDS. Using child death review board files, Welch and Bonner (2013) studied patterns of fatal child neglect in Oklahoma over 22 years. AI/AN children were disproportionately represented in the sample of deaths, with most due to supervisory/environmental neglect leading to unintentional drowning and smoke inhalation. While AI/AN accounted for approximately 9% of Oklahoma’s population, AI/AN children represented 13% of victims of fatal neglect.

Data from national surveys also document higher rates of physical abuse in AI/AN as compared to the general population. The National Survey of Adolescents determined that lifetime prevalence of childhood physical abuse among AI/AN youth is 15%, second only to African-American youth at 16% (Hanson et al. 2006) (Table 1). Hawkins et al.’s (2010) national survey of 12 to 17-year-olds found a similar prevalence of child physical abuse in AI/AN and African Americans (15%).

**Table 1:** **Prevalence of childhood abuse and neglect in American Indians/Alaska natives, by year of study**

First author year	Population and data source	Sample size	Measure	Prevalence
Lodico et al. 1996	9^th^ and 12^th^ grade students of a Midwestern state; 10% random sample of all white, African American, and AI/AN respondents with anonymous self-report survey of risk behaviors	494 AI/AN adolescents	Lifetime sexual abuse by family	2%
			Lifetime sexual abuse by nonfamily members	10%
			Lifetime sexual abuse by both family and nonfamily members	6%
			Lifetime any form of sexual abuse both genders	17%
			Lifetime any form of sexual abuse in males	8%
			Lifetime any form of sexual abuse in females	28%
Robin et al. 1997	Southwestern tribe; enrolled tribal members over age 21; semi-structured psychiatric interview	582 AI/AN adults	Childhood sexual abuse occurring before 16 years of age in women	49%
		≥21 years of age		
			Childhood sexual abuse occurring before 16 years of age in men	14%
Saewyc et al. 2003	2 cohorts (1992, 1998) of 9^th^ and 12^th^ graders in Minnesota public schools completing state-wide anonymous self-report survey of risk behavior	1992: 750 AI/AN adolescents	Lifetime sexual abuse by family	3%
			Sexual abuse by nonfamily members	8%
			Lifetime prevalence of sexual abuse by both family and nonfamily members	7%
			Lifetime any form of sexual abuse	18%
		1998: 548 AI/AN adolescents	Lifetime sexual abuse by family	3%
			Lifetime sexual abuse by nonfamily member	10%
			Lifetime sexual abuse by both family and nonfamily members	4%
			Lifetime any form of sexual abuse	17%
Duran et al. 2004	Outpatient primary care clinic at community-based Indian Health Hospital in Albuquerque, clinic-based sampling with self-report survey	234 AI/AN women age 18-45	At least one type of maltreatment in childhood	77%
			Childhood neglect	63%
			Childhood emotional abuse	55%
			Childhood sexual abuse	44%
			Childhood physical abuse	42%
			Childhood sexual and physical abuse	28%
Libby et al. 2005	Southwestern tribe and 2 closely affiliated Northern plains tribes; stratified random sampling of tribal rolls computer-assisted in-person interviews	*Southwest tribe*	Childhood physical abuse before age 13 in males	7%
		1,446 Southwest tribal members age 15-54	Childhood sexual abuse before age 13 in males	2%
			Childhood physical abuse before age 13 in females	7%
			Childhood sexual abuse before age 13 in females	8%
			Childhood physical abuse before age 13 in both males and females males	7%
			Childhood sexual abuse before age 13 in both males and females	5%
		*Northern Plains tribes*	Childhood physical abuse before age 13 in males	7%
		1,638 Northern Plains tribal members age 15-54 (3,084 AI/AN participants total)	Childhood sexual abuse	1%
			Childhood physical abuse before age 13 in females	9%
			Childhood sexual abuse before age 13 in females	7%
			Childhood physical abuse before age 13 in both males and females	8%
			Childhood sexual abuse before age 13 in both males and females	4%
Hanson et al. 2006	U.S. National household probability sample of 12-17 year olds with self-report survey	139 AI/AN adolescents	Lifetime physically abusive punishment by caretakers	15%
			Lifetime intrafamilial sexual abuse	4%
			Lifetime extrafamilial sexual abuse	11%
Saylors and Daliparthy 2006	Residential and outpatient substance abuse treatment settings in urban Oakland and San Francisco, California; self-report survey	283 AI/AN women in treatment	Childhood sexual abuse	56%
			First incident at ages 1-5	37%
			First incident at ages 6-10	37%
de Ravello et al. 2008	New Mexico prison system; self-report survey	36 AI/AN incarcerated women age 20-60 years	Childhood sexual abuse	53%
			Childhood physical abuse	42%
			Childhood physical and sexual abuse	33%
			Childhood physical neglect	22%
Hawkins et al. 2010	U.S. National household probability sample of 12-17 year olds with self-report survey	86 AI/AN adolescents	Lifetime physical abuse by caretakers	15%
Welch and Bonner 2013	Oklahoma death review board files of fatalities attributed to child neglect; administrative data	47 AI/AN child death cases	Fatality due to neglect	13%

*Abbreviation*: *AI/AN* American Indian/Alaska Native.

Studies using non-national sampling frames suggest that rates of child abuse and neglect may be higher than officially reported or captured in national surveys. In a cross-sectional study of 234 AI/AN women at an Indian Health Service (IHS) outpatient clinic, 77% of respondents reported some type of childhood abuse or neglect (Duran et al. 2004). Neglect was the most common maltreatment in this sample, with 63% experiencing physical and/or emotional neglect. The study found that women reporting neglect were also much more likely to have experienced abuse, with approximately 90% of the women neglected in childhood also experiencing physical, emotional, or sexual abuse. In another sample of 36 AI/AN incarcerated women, 42% reported that they were physically abused by family members, with 33% reporting both sexual and physical abuse by family members or loved ones (de Ravello et al. 2008). However, one relatively large study involving a Southwestern tribe and two Northern Plains tribes reported childhood physical abuse prevalence ranging from 7% to 9%, a figure much lower than other studies (Libby et al. 2005).

Reports of childhood sexual abuse among AI/AN populations are common. Seventeen percent of AI/AN adolescents in Minnesota schools reported at least one instance of childhood sexual abuse (Saewyc et al. 2003). Forty-nine percent of women and 14% of men in a Southwestern tribe reported experiences of childhood sexual abuse (Robin et al. 1997). In de Ravello et al.’s (2008) sample of 36 incarcerated AI/AN women, 53% were sexually abused by family members or loved ones. Libby et al. (2005)’s study of the Southwestern and Northern Plains tribes found sexual abuse prevalence to range from 1% to 8%.

In another study of women receiving treatment for substance use and mental illness in an urban area, abuse started early, with 37% of women reporting sexual abuse beginning at ages 1-5 years and another 37% reporting first experiences at ages 6-10 years (Saylors and Daliparthy 2006). In a state-wide study of school-based adolescents, AI/AN respondents were 1.8 times more likely than white respondents to report childhood sexual abuse (Lodico et al. 1996). The same study showed that 17% of AI/AN students, compared to 9% of white students, reported childhood sexual abuse.

### Prevalence of violence against women

Violence against women encompasses both intimate partner violence (IPV) and sexual violence (World Health Organization 2013). While rape, forced sex, sexual violence, and sexual abuse have distinct definitions, our discussion of sexual violence covers all of these types of violence. Prevalence estimates for each type are presented as described within individual studies. The prevalence of VAW among AI/AN is among the highest for all races/ethnicities, second only to multiracial women. In the nationally representative National Intimate Partner and Sexual Violence Survey (NISVS), 27% of AI/AN women reported lifetime rape, higher than prevalence for black (22%), white (19%), and Hispanic (15%) women, though lower than multiracial (34%) women (Black et al. 2011) (Table 2). Seventeen percent of AI/AN women report that their first sexual encounter was forced (Rutman et al. 2012) compared with 10% of the general American female population in a national survey (Gavin et al. 2009). In NISVS, 46% of AI/AN reported lifetime physical assault by intimate partner, also higher than prevalence for black (41%), white (32%), and Hispanic (35%) women, though lower than multiracial (50%) women (Black et al. 2011).

**Table 2:** **Prevalence of violence against women among American Indians/Alaska natives, by year of study**

First author year	Population and data source	Sample size	Measure	Prevalence
Curry 1998	Prenatal clinics in a Northwest city; clinic-based sample with self-report survey	57 AI/AN women	Past year physical IPV	37%
			Past year sexual IPV	7%
			IPV in pregnancy	12%
Fairchild et al. 1998	Indian health service facility on a Navajo	341 AI/AN women	Lifetime IPV	53%
	Reservation; clinic-based sample with self-report survey		Lifetime physical IPV	42%
			Lifetime sexual IPV	12%
			Past year IPV	16%
			Past year physical IPV	14%
			Past year sexual IPV	4%
Kvigne et al. 1998	Pregnant women from a Northern plains tribe; clinic-based sample with self-report survey	177 AI/AN women	IPV in pregnancy	30%
Robin et al. 1998	Southwestern tribe; referral-based sampling with self-report survey	56 married	Lifetime IPV	91%
		AI/AN women	Lifetime physical IPV	79%
			Lifetime sexual IPV	29%
			Past year IPV	31%
			IPV in pregnancy	34%
			IPV injuries requiring medical attention	46%
Bohn 2002	Prenatal clinic in Midwestern city; clinic-based sample with self-report survey	30 AI/AN women	IPV in pregnancy	33%
			Threats to kill woman or her baby in pregnancy	6%
Bohn 2003	Prenatal clinic in a Midwestern city; clinic-based sample with self-report survey	30 AI/AN women	Lifetime IPV	87%
			Current partner IPV	70%
			Current partner physical IPV	60%
			Current partner sexual IPV	17%
Harwell et al. 2003	Seven reservations in Montana; random digit dial sample with self-report survey	588 AI/AN women	Past year physical IPV	3%
Malcoe et al. 2004	WIC clinic in Oklahoma; clinic-based sample with self-report	312 AI/AN women	Lifetime IPV	59%
			Lifetime sexual IPV	12%
			Lifetime threats with knife/gun	11%
			Past year IPV	30%
			Past year sexual IPV	3%
			IPV in pregnancy, past year	9%
			IPV resulting in injuries	40%
Simoni et al. 2004	New York City AI/AN community center members; self-report survey	155 AI/AN women	Lifetime rape	34%
			Lifetime sexual IPV	20%
			Lifetime physical IPV	31%
Manson et al. 2005	2 Northern plains tribes and 1 Southwestern tribe; random sampling from tribal rolls with self-report survey	829 AI/AN women in Southwest	Lifetime rape, Southwest	13%
		848 AI/AN women in Northern plains	Lifetime rape, Northern plains	14%
			Lifetime physical IPV, Southwest	29%
			Lifetime physical IPV, Northern plains	31%
Evans-Campbell et al. 2006	New York City AI/AN; respondent-driven, chain referral, and targeted sampling with self-report survey	112 AI/AN women	Lifetime IPV	40%
			Lifetime rape	48%
Yuan et al. 2006	Six tribes in the Southwest, Northwest, Northern plains, and Northeast; random sample from tribal rolls with self-report survey	744 AI/AN women	Lifetime rape	14%
Mylant and Mann 2008	Teenage mothers in the Northern plains participating in pregnancy program; self-report survey	49 AI/AN women	Lifetime IPV	61%
			IPV in pregnancy	38%
			Past year sexual IPV	23%
Duran et al. 2009	Indian health services clinic in Albuquerque; clinic-based sample with self-report survey	324 AI/AN women	Lifetime IPV	80%
Wood and Magen 2009	Athabaskan tribe, Alaska; self-report survey	91 AI/AN women	Lifetime IPV	64%
			Past year physical IPV	18%
			Lifetime threats with gun	19%
			Lifetime threats with knife	12%
Rutman et al. 2012	National survey with self-report	253 AI/AN women	Forced first sexual encounter	17%
Scott and Langhorne 2012	Boarding school for at-risk AI/AN youth in a plains state; self-report survey	115 AI/AN women	Lifetime rape	20%
			Lifetime IPV	70%
Ehlers et al. 2013	Eight contiguous reservations in California; respondent-driven and venue based sampling with self-report survey	174 AI/AN women	Lifetime sexual abuse	34%
Black et al. 2011	National survey with self-report	Not reported	Lifetime rape	27%
			Lifetime physical IPV	46%

*Abbreviations*: *AI/AN* American Indian/Alaska Native, *IPV* intimate partner violence.

Studies conducted among various tribal communities, urban AI/AN populations, and health care facilities serving AI/AN provide evidence to support the high prevalence of lifetime VAW reported in NISVS. Lifetime prevalence of rape ranged from 4%-29% among women from six geographically diverse tribes in Yuan et al’s (2006) Ten Tribes study to 48% among AI/AN women in New York City (Evans-Campbell et al. 2006). Among young women attending boarding school for at-risk AI/AN, 20% reported forced sex (Scott and Langhorne 2012), and lifetime prevalence of sexual abuse among women living on reservations in California was 34% (Ehlers et al. 2013). Lifetime prevalence of sexual violence in intimate partner relationships ranged from 12% among women in IHS health care (Fairchild et al. 1998) to 29% among women in a Southwestern tribe (Robin et al. 1998). Lifetime physical assault by an intimate partner ranged from 29% among AI/AN women living on a reservation in the Southwest (Manson et al. 2005) to 79% among women in a Southwestern tribe (Robin et al. 1998) with several estimates between 30-40% (Fairchild et al. 1998; Simoni et al. 2004; Manson et al. 2005). Lifetime prevalence of any kind of IPV is consistently high across studies, ranging from 40% among AI/AN women in New York City (Evans-Campbell et al. 2006) to 91% among married women in a Southwestern tribe (Robin et al. 1998), with most estimates greater than 50% (Fairchild et al. 1998; Bohn 2003; Malcoe et al. 2004; Mylant and Mann 2008; Duran et al. 2009; Wood and Magen 2009).

Prevalence estimates for recent experiences of IPV vary but are as high as 60%. Physical abuse in the past year was reported by 3% of women living on Montana reservations (Harwell et al. 2003), 14% of women presenting for care at an IHS health facility (Fairchild et al. 1998), and 37% of pregnant women in a Northwest city (Curry 1998). Among pregnant women receiving care at a Midwestern Indian clinic, 60% reported physical assault by their current partner (Bohn 2003). Sexual abuse in the past year was reported by 3% of women receiving services at a WIC clinic (Malcoe et al. 2004), 9% of currently pregnant women (Curry 1998), and 23% of teenage mothers (Mylant and Mann 2008).

IPV is common among AI/AN women during pregnancy. In several studies, one in three AI/AN women report IPV in pregnancy (Kvinge et al. 1998; Robin et al. 1998; Bohn 2002) with estimates ranging from 9% among AI/AN women pregnant in the last year (Malcoe et al. 2004) to 38% among teenage AI/AN mothers (Mylant and Mann 2008). In one survey, 6% of currently pregnant women reported that their partner had threatened to kill them or their baby (Bohn 2002).

Weapons are more frequently associated with IPV incidents among AI/AN women than among the general population of American women. Among Alaska Native women, 19% were threatened with a gun and 12% were threatened with a knife in their lifetimes (Wood and Magen 2009). Among AI/AN women attending a WIC clinic in Oklahoma, 11% of women had been threatened with a knife or gun (Malcoe et al. 2004). This is in comparison with 6% of the general population of American women who were threatened with a gun and 6% who were threatened with a knife (Tjaden and Thoennes 2000). Injuries perpetrated by partners were common among women attending WIC clinics (40%; Malcoe et al. 2004) and married women in a Southwestern Tribe (46%%; Robin et al. 1998).

Rates of femicide in the AI/AN population are also very high. The rate of domestic violence homicide in New Mexico from 1990-1993 among AI/AN women was 4.9/100,000 women, which was significantly higher than the rates for Hispanics (1.7/100,000) and non-Hispanic whites (1.8/100,000) (Arbuckle et al. 1996). Firearms were used twice as often in domestic violence homicides compared to homicides not related to domestic violence (Arbuckle et al. 1996).

### Prevalence of elder abuse

Variation in definition and terminology is a challenge in elder abuse research (National Research Council 2003). The Research Council defines elder abuse as "any knowing, intentional, or negligent act by a caregiver or any other person that causes harm or a serious risk of harm to a vulnerable adult.” This paper does not confine itself to a particular definition of abuse, and uses "elder abuse" to refer to various kinds of mistreatment, exploitation and neglect. Estimates of elder abuse prevalence are widely variable in the few studies that have explored this issue. Drawing on diverse studies of AI/AN populations, Buchwald et al. (2000) found prevalence estimates of abuse ranging from 2% to 46% among AI/AN populations (Minton and Soule 1990; John 1995 in Buchwald et al. 2000) (Table 3). In an analysis of 550 medical charts of urban AI/AN, Buchwald et al. (2000) found 10% prevalence of probable or definite physical abuse. This was similar to rates of physical abuse found in several smaller studies of AI/AN elders: 11% of Alaska Natives (Minton and Soule 1990), 16% of Navajos (Brown 1989), and 19% of Northern Cheyennes (Cheyenne 1993 in Buchwald et al. 2000). In a cross-cultural study of the occurrence of elder abuse among seven cultural groups, Hudson et al. (1998) found that 4% of AI/AN surveyed reported abuse occurring after age 65. This rate was lower than for other racial groups, although the experience of abuse over the lifetime was highest for AI/AN (26%).

**Table 3:** **Prevalence of elder abuse among American Indians/Alaska natives, by year of study**

First author year	Population and data source	Sample size	Measure	Prevalence
Brown 1989	Navajo tribe, Oljato chapter; randomly selected tribal members with self-report survey.	Random sample of 37 AI/AN elderly from a population of 110 AI/AN elderly	Reported some extent of having been left alone and neglected when they needed help	32%
			Financially exploited by family members	22%
			Neglected in some way	46%
			Psychologically abused	22%
			Physically abused	16%
Hudson et al. 1998	Two tribal groups in North Carolina; random sample of elderly tribal members with self-report survey.	200 AI/AN;	Been abused at some time in life (all ages)	26%
		92 over 65-years-old	Abused after the age of 65	4%
Buchwald et al. 2000	Chart review of urban AI/AN in primary care in King County; clinic-based sample with medical chart review.	550 AI/AN elderly	Definite or probable physical abuse	10%
		age ≥50	Suggestive physical abuse	7%
Minton and Soule 1990	216 Alaska natives from two rural Alaskan villages; randomly selected tribal members with structured interview responses.	52 AI/AN elderly	Reported sadness as a result of victimization	11%
		age ≥55		

*Abbreviation*: *AI/AN* American Indian/Alaska Native.

Despite limited data on prevalence, tribal leaders express significant concern about elder abuse (National Indian Council on Aging 2004; Brown 1989; Maxwell and Maxwell 1992). In a survey conducted by the Office of Aging Americans of Tribal Title VI directors, 48% perceived that elder neglect occurred often and 39% that psychological or verbal abuse occurred often. This abuse was perceived to occur most often at the hand of spouses/partners and other family members (Jackson and Sappier 2005).

Though there is a high variability of prevalence estimates, it is clear that when elder abuse does occur, it is rarely officially reported. Buchwald et al. (2000) found that only 31% of definite cases of abuse of elderly AI/AN were reported to authorities. This low rate of reporting is consistent with rates of reporting in other elderly populations in the United States. The National Elder Abuse Incidence Study estimated that only 18% of cases of elder abuse and neglect occurring in domestic settings are reported to adult services (American Public Human Services Association 1998).

### Risk factors for interpersonal violence

Many of the risk factors for interpersonal violence identified among AI/AN are not unique to this population. AI/AN risk factors for child abuse and neglect, such as non-married parental status, parental substance abuse, parental history of abuse, parental unemployment, greater number of children in the home, low SES, disabilities in the child, and female gender, are also identified risk factors in other populations (reviewed in Putnam 2003; Sedlak et al. 2010). Low income, low educational attainment, unemployment, younger age, non-married status, childhood abuse, and alcohol use are consistent risk factors for VAW in AI/AN and other populations (reviewed in Capaldi et al. 2012). Risk factors for violence against the elderly include dementia and social isolation in the victims and mental disorders and alcohol misuse among perpetrators as noted in studies of AI/AN and other racial/ethnic groups (reviewed in Lachs and Pillemer 2004).

### Common risk factors across the life course

A number of factors have been identified that increase the risk of abuse and neglect in AI/AN at every stage of life. Child abuse and VAW have several common risk factors including non-married partnership, female gender of the victim, and substance abuse by the perpetrator (DeBruyn et al. 1992; Horejsi et al. 1992; Nelson et al. 1996; Piasecki et al. 1989; Robin et al. 1997; White and Cornely 1981; Malcoe et al. 2004; Yuan et al. 2006; Robin et al. 1998). While the exact mechanism between alcohol/substance use and perpetration of child neglect/abuse/VAW is unclear, several hypotheses have been suggested (Walsh et al. 2003; Kunitz et al. 1998). Alcohol and other drugs impair mental functioning, judgment, and protective tendencies of users by suppressing brain centers that inhibit socially unacceptable behaviors, leading to perpetration of VAW and child abuse (Goldman et al. 2003; Walsh et al. 2003; Kunitz et al. 1998; Yuan et al. 2006). Caregivers and partners with severe substance dependence may also experience economic difficulties secondary to their alcohol/substance use, compromising their ability to provide for those under their care (Goldman et al. 2003).

Researchers have also proposed societal factors that may increase the prevalence of these risk factors, especially with respect to substance abuse (DeBruyn et al. 2001). Important sociocultural contexts that might affect this include high rates of unemployment and poverty, governmental oppression, racism, under-education, erosion of cultural and traditional lifestyles, and widening chasms between generations (BigFoot and Schmidt 2010; Cross et al. 2000; DeBruyn et al. 2001; DeBruyn et al. 1992; Fischler 1985; Fox 2003; Nelson et al. 1996). Lack of resources, including low income and unemployment, are also common risk factors for perpetration of child and elder abuse and VAW across populations (Maxwell and Maxwell 1992; Malcoe et al. 2004).

### Risk factors of childhood abuse

Several individual and interpersonal risk factors for childhood abuse and neglect have been identified. Certain family structure factors increase the likelihood of child maltreatment among AI/AN, including divorce, teenage parenthood, having children outside of marriage, and single parenthood (DeBruyn et al. 1992; Horejsi et al. 1992; Piasecki et al. 1989; White and Cornely 1981). Other risk factors for experiencing child abuse and neglect include children switching homes (DeBruyn et al. 1992), having multiple men associated with the household (Nelson et al. 1996), stressful life events in the household (Nelson et al. 1996), and having a greater number of children in the household (Horejsi et al. 1992; Nelson et al. 1996; White and Cornely 1981). The presence of psychiatric problems in the caregiver (Nelson et al. 1996), caregiver criminal records (DeBruyn et al. 1992; Lujan et al. 1989; Nelson et al. 1996), and/or of childhood abuse/domestic violence in caregivers’ lives (Berlin 1987; Cross et al. 2000; DeBruyn et al. 1992; Fernandez 1987; Horejsi et al. 1992; Lujan et al. 1989; Mannes 1993) are risk factors for perpetrating abuse and neglect. Although child disability is associated with maltreatment, the direction of the effect in AI/AN remains unclear (DeBruyn et al. 1992; Fischler 1985; U.S. Dept of Health and Human Services 2010).

### Risk factors of violence against women

Women experiencing childhood sexual or physical abuse are also more likely to experience physical and sexual assault in adulthood (Bohn 2003; Yuan et al. 2006). Exposure to maltreatment in childhood is a risk factor for alcohol and substance dependence and other mental disorders later in life for AI/AN women (de Ravello et al. 2008; Duran et al. 2004; Koss et al. 2003). The elevated rates of substance dependence may act as a mediator for increased prevalence of VAW in women with history of child abuse and neglect.

Alcohol use by both victims and perpetrators has been identified as a risk factor for VAW. Lifetime alcohol dependence was a risk factor for both physical and sexual assault among women in the Ten Tribes study (Yuan et al. 2006). Seventy-four percent of IPV in a Southwestern tribe involved alcohol use by either the perpetrator or the victim (Robin et al. 1998). Alcohol or drug use immediately prior to death was also common (54%) among femicide victims in New Mexico in the early 1990s (Arbuckle et al. 1996).

Several demographic factors are associated with VAW among AI/AN. For example, lower educational attainment and unemployment among women have been associated with lifetime experiences of physical assault (Harwell et al. 2003). Socioeconomic status is also an important factor; among women attending a WIC clinic, women with low socioeconomic SES (defined as living at or below 50% of the poverty line, receiving other government assistance, or having a partner with less than a high school education) had a past year IPV prevalence of 43% compared with 10% among women receiving services at WIC who did not meet the low SES criteria (Malcoe et al. 2004) (Table 4). Similarly, Navajo women living in a household that received government assistance had twice the odds of experiencing IPV in the past year (Fairchild et al. 1998). Partner’s lower educational attainment and unemployment were also associated with past year IPV in bivariable but not multivariable analyses, after adjusting for low SES among women attending a WIC clinic (Malcoe et al. 2004). Younger age (Fairchild et al. 1998; Harwell et al. 2003; Malcoe et al. 2004) and being separated/divorced or cohabiting with a partner were also associated with past year IPV in several studies (Malcoe et al. 2004; Yuan et al. 2006). Pregnancy is also a period of vulnerability to VAW (Kvinge et al. 1998; Robin et al. 1998; Bohn 2002; Malcoe et al. 2004; Mylant and Mann 2008).

**Table 4:** **Risk factors for interpersonal violence in American Indian/Alaska native children, women, and elders, by violence type**

First author year	Population	Sample size	Risk factor	Outcome
*Childhood abuse*				
White and Cornely 1981	Records Navajo children <9 years old	365 abused and/or neglected AI/AN children	Unmarried parents	Abuse or neglect
		867 comparison AI/AN children	Number of children in household	Abuse or neglect
Lujan et al. 1989	Southwestern Indian health service hospital	117 medical records of abused and/or neglected AI/AN children ages 1-21	Alcohol abuse by caretaker	Abuse or neglect
			History of abuse or neglect in caretaker	Abuse or neglect
			Disability in child	Abuse or neglect
Nelson et al. 1996	Mesquakie tribe in Tama Country, Iowa; Siletz and other Northwest tribes in 11-county area in Northern Oregon	39 neglecting AI/AN families	Mother under 19 years of age at first birth	Neglect
		38 comparison AI/AN families	More than 1 father associated with household	Neglect
			Number of children in household	Neglect
			Parents separated or divorced	Neglect
			Substance abuse in caretaker	Neglect
			Criminal charges on caretaker’s record	Neglect
			Caretaker has or is receiving psychiatric treatment	Neglect
*Violence against women*				
Arbuckle et al. 1996	Female homicide victims in New Mexico	33 AI/AN women	Alcohol or drug use by victim	Homicide by partner
Fairchild et al. 1998	Indian health service facility on a Navajo reservation	341 AI/AN women	Receiving government assistance	Current IPV
			Age (under 40)	Current IPV
Robin et al. 1998	Southwestern tribe	56 married AI/AN women	Alcohol use by either perpetrator or victim	Lifetime IPV
Bohn 2003	Clinic in a Midwestern city	30 AI/AN women	Childhood abuse	Adult abuse
Harwell et al. 2003	Seven reservations in Montana	588 AI/AN women	Age (under 45)	Past year physical IPV
Malcoe et al. 2004	WIC clinic in Oklahoma	312 AI/AN women	Low SES (living at or below 50% poverty line; receiving government assistance; or partner with < HS education)	Past year IPV
			Age (under 32)	Past year IPV
			Separation/divorce	Past year IPV
Yuan et al. 2006	Six tribes in the Southwest, Northwest, Northern plains, and Northeast	744 AI/AN women	Alcohol dependence	Physical assault
				Sexual assault
			Cohabitation	Physical assault
				Sexual assault
			Separation/divorce	Physical assault
				Sexual assault
			Childhood sexual abuse	Sexual assault
*Elder abuse*				
Brown 1989	One Navajo tribe, Oljato chapter (110 total elders)	Random sample of 37 elderly and one family member of each elder	Suddenness of onset of dependence on family	Abuse overall
			Family perception of dependency	Abuse overall
				Neglect
			Mental condition less than normal, as perceived by family	Abuse overall
Maxwell and Maxwell 1992	2 Plains Indians tribes	Community-wide ethnographic study	Caregivers who abused tended to be younger and live with their elders; abuse was more common among less wealthy tribe	N/A
Buchwald et al. 2000	Chart review of urban AI/AN (age >50) in primary care in King County	550 AI/AN elderly	Younger age	Definite or probable physical abuse
			Female	Definite or probable physical abuse
			Currently depressed	Definite or probable physical abuse
			More likely to depend on	Definite or probable physical abuse
			others for food	

*Abbreviations*: *AI/AN* American Indian/Alaska Native, *IPV* intimate partner violence, *OR* odds ratio.

### Risk factors of elder abuse

Risk factors for elder abuse in AI/AN populations include gender, disability, and pace of worsening cognitive impairment (Lachs et al. 1994, 1997), and have been suggested to include poverty and a variety of its concomitants: poor health and high rates of illness; frequent loss and trauma; high rates of mental health issues; physical disability; drug and alcohol abuse, and others (Carson 1995). Dependency and inter-dependency among elderly AI/AN and their adult children is common, and among urban AI/AN elders, Buchwald et al. (2000) found significant associations between physical abuse and younger age, female sex, current depression, and dependence on others for food. However, among elderly Navajo, Brown (1989) noted a relationship between abuse and elders having an income.

In the most comprehensive ethnographic study conducted on elder abuse, Maxwell and Maxwell (1992) found that caregiver characteristics, specifically isolation and lack of access to resources, were related to abuse. They conducted comparative work in two Plains tribes, taking a resource-based approach to understanding the contexts in which abuse occurs. They found that sexual abuse was limited to the more isolated and resource-poor reservation, and that rates of abuse overall were also higher at this site, with intentional abuse occurring more frequently on the resource-poor reservation. In a survey in a Plains community, Maxwell and Maxwell (1992) found that the caregivers who abused tended to be younger and to live with their elders. In a survey of a Navajo community, Brown (1989) found that elder abuse was associated especially with caregivers with personal problems, and for elders, a suddenness of onset of dependence on family, elders having mental problems, and family crises as a result of providing care suddenly, for which they were not prepared.

### Prevalence of risk factors among AI/AN

While AI/AN share many of the same risk factors for interpersonal violence as other populations, AI/AN have a much higher prevalence of these risk factors. The AI/AN population has a different demographic profile than the general U.S. population, which includes a higher prevalence of several factors associated with interpersonal violence. Birth rates are higher among young AI/AN women than for young women overall (DeVoe and Darling-Churchill 2008), and AI/AN women were more likely to have given birth during the last year than non-Hispanic Whites (U.S. Census Bureau 2004). AI/AN people are also more likely to be divorced or never married than non-Hispanic Whites. In 2004, 42% of AI/AN aged 15 or older were married, compared to 57% of non-Hispanic Whites (U.S. Census Bureau 2004).

Economic disadvantage is high among AI/AN populations. The AI/AN poverty rate (26%) is twice the national rate, and AI/AN fail to complete high school at a higher percentage (29%) than the nation overall (Ogunwole 2006). AI/AN unemployment rates were among the highest for all racial/ethnic groups at 15%, compared to 9% average unemployment rate across all groups (DeVoe and Darling-Churchill 2008).

Studies document that AI/AN suffer higher rates of alcohol-related morbidity and mortality than their non-AI/AN counterparts. They are more likely to experience alcohol and illicit drug use disorders (Substance Abuse and Mental Health Services 2010) and die from alcohol-attributable deaths (Centers for Disease Control and Prevention 2008). In two national samples of adolescents, AI/AN youth had initiated alcohol and substance use earlier (Wallace et al. 2003), were more likely to have used alcohol or other drugs in the past 30 days (Wallace et al. 2003), and to have alcohol or drug dependence (Wu et al. 2011) than any other racial/ethnic group. Past year binge drinking is 30% in AI/AN adults, higher than any other racial/ethnic group (Substance Abuse and Mental Health Services 2008).

### Limitations in existing research

One of the greatest limitations in interpersonal violence research among AI/AN is the scarcity of valid prevalence estimates. To obtain these estimates, sufficient sample sizes and proper sampling frames are necessary. Limited investigation into risk and protective factors for interpersonal violence and lack of a life course perspective in understanding the interconnectedness of interpersonal violence are other important limitations.

A special challenge in researching abuse and neglect in AI/AN populations are cultural differences in the definitions of abuse and neglect ascribed by AI/AN and non-AI/AN people. Some researchers in the area caution that neglect may be over-reported by non-AI/AN reporters of child maltreatment due to misunderstanding of the AI/AN communal child rearing system (Earle and Cross 2001; Fox 2003; Fischler 1985). Another challenge to accurate prevalence estimates is that, for cultural reasons, autopsies are rarely conducted on AI/AN children who present dead on arrival at the hospital, potentially leading to undercounting of fatal child abuse and neglect.

Measuring the prevalence of elder abuse is complicated by cultural differences in conceptualizing abuse. In the few cross-cultural studies conducted, AI/AN populations tend to rate more behaviors as abusive, and more seriously abusive, than other cultures (Hudson and Carlson 1999). Among Navajo social service providers, those providers with the highest degree of identification with Navajo cultural values had a greater likelihood of perceiving elder abuse and neglect as problematic (Brown 1989). These findings highlight the need to construct valid instruments to measure abuse that permit meaningful comparisons across cultures.

Generalizability across studies of AI/AN violence is limited. AI/AN populations are culturally diverse, with more than 560 federally recognized tribes (Bureau of Indian Affairs 2011). They are also geographically diverse, with 60% of affiliated AI/AN residing off tribal lands (Ogunwole 2006). Additionally, much of the research on violence in AI/AN communities is more than a decade old and may not reflect the current experiences interpersonal violence in these populations.

### Recommendations for future research

Given the public health importance of interpersonal violence in AI/AN populations, more research is needed in order to develop culturally acceptable prevention and treatment strategies. In particular, new research methods may be necessary to provide important information on prevalence, risk and protective factors, and effectiveness of interventions. More cross-tribal and nationally representative surveys are necessary to understand the larger burden of interpersonal violence among AI/AN. Nationally representative surveys will need to employ oversampling procedures; this will ensure AI/AN sample sizes sufficient to produce stable prevalence estimates and to facilitate statistical analysis of risk and protective factors. Additionally, long term follow-up studies are needed to understand the interconnectedness of interpersonal violence across the life course for AI/AN populations, including common risk and protective factors and changing vulnerabilities from childhood through later life. Research involving AI/AN populations should be culturally sensitive, mindful of past injustices, and cognizant of the metholodological challenges in measuring interpersonal violence.

Given cultural differences in understandings of child and elder abuse, research in abuse and neglect would benefit from standardization of definitions and measurements. Survey instruments must be culturally sensitive so that meaningful constructs of maltreatment can be documented. Coordination and consistency among researchers working in these areas will be instrumental in creating comprehensive information on abuse among AI/AN populations. There may also be an important role for ethnographic studies in this area.

Conducting research among AI/AN populations requires many special considerations. Tribal leaders should be involved in the establishment of any research projects as tribal institutional review boards or community advisory boards may need to approve research (Katz et al. 2011; Yuan et al. 2006). Confidentiality requirements may extend beyond protecting individual confidentiality to maintaining the confidentiality of the tribe (Katz et al. 2011; Yuan et al. 2006). Research among AI/AN must begin with a plan to relay findings to the community in a timely fashion (Allen et al. 2006). Because data on tribal members’ experiences are nested within the cultural context of the tribe, misinterpretation by outsiders may occur. To minimize this, investigators may want to consider including co-researchers from the tribe (Allen et al. 2006), and having any documents intended for wide dissemination first approved by tribal representatives (Holkup et al. 2004). The selection of interviewers for sensitive topics such as abuse must be carefully considered. In the Ten Tribes study, while all interviews were conducted by AI/AN, tribes that used their own members as interviewers reported lower prevalence of interpersonal violence than tribes using interviewers from other nations (Yuan et al. 2006).

Another potentially useful approach to research involving AI/AN is community-based participatory research (CBPR), as it is designed to explicitly acknowledgeand minimize the potential mistrust of outside researchers, a common problem among AI/AN communities. CBPR approaches address many of the concerns noted above by insuring the full participation and inclusion of tribal members in all aspects of the research project (Holkup et al. 2004). CBPR is characterized by working with the community as equal and full partners in the development, implementation, and evaluation of interventions, and the sharing of any knowledge gained with the community (Daley et al. 2010; Holkup et al. 2004). CBPR has been successfully used among AI/AN populations for other health issues (Chester et al. 1999; Daley et al. 2010; Holkup et al. 2007; Johnson et al. 2007; King 1999; Salois et al. 2006) and may be useful for studies on interpersonal violence.

## Conclusions

Anecdotal, survey-based and field study research consistently shows high prevalence of family and partner interpersonal violence in AI/AN populations. Child abuse and neglect, particularly childhood sexual abuse, are highly prevalent among AI/AN, as are experiences of VAW. Elder abuse and neglect are a concern among AI/AN populations, though prevalence estimates are few. While risk factors for violence in this population are not unique, they are also highly prevalent, contributing to the high burden of interpersonal violence in AI/AN communities. Future work among AI/AN should be culturally sensitive, provide valid estimates of prevalence, identify risk and protective factors, and consider the interconnectedness of interpersonal violence across the life course.

## Authors’ original submitted files for images

Below are the links to the authors’ original submitted files for images. Authors’ original file for figure 1 Authors’ original file for figure 2 Authors’ original file for figure 3 Authors’ original file for figure 4
