# Supplementary material for: Family and partner interpersonal violence among American Indians/Alaska Natives
Source: Inj Epidemiol. 2014 Mar 20;1(1):7. doi: 10.1186/2197-1714-1-7 (PMC5005741; doi:10.1186/2197-1714-1-7)
Supplement: Supplementary file 1 — Authors’ original file for figure 1 [file 40621_2013_7_MOESM1_ESM.doc]

Table 1. Prevalence of childhood abuse and neglect in American Indians/Alaska Natives, by year of study

| First Author Year | Population and Data Source | Sample Size | Measure | Prevalence |
| --- | --- | --- | --- | --- |
| Lodico 1996 | 9^th^ and 12^th^ grade students  of a Midwestern state; 10% random sample of all white, African American, and AI/AN respondents with anonymous self-report survey of risk behaviors | 494 AI/AN adolescents | Lifetime sexual abuse by family | 2% |
|  |  |  | Lifetime sexual abuse by nonfamily members | 10% |
|  |  |  | Lifetime sexual abuse by both family and nonfamily members | 6% |
|  |  |  | Lifetime any form of sexual abuse both genders | 17% |
|  |  |  | Lifetime any form of sexual abuse in males | 8% |
|  |  |  | Lifetime any form of sexual abuse in females | 28% |
| Robin 1997 | Southwestern Tribe; enrolled tribal members over age 21; semi-structured psychiatric interview | 582 AI/AN adults ≥21 years of age | Childhood sexual abuse occurring before 16 years of age in women | 49% |
|  |  |  | Childhood sexual abuse occurring before 16 years of age in men | 14% |
| Saewyc 2003 | 2 cohorts (1992, 1998) of  9^th^ and 12^th^ graders in Minnesota public schools completing state-wide anonymous self-report survey of risk behavior | 1992: 750 AI/AN adolescents | Lifetime sexual abuse by   family | 3% |
|  |  |  | Sexual abuse by  nonfamily members | 8% |
|  |  |  | Lifetime prevalence of  sexual abuse by both  family and nonfamily  members | 7% |
|  |  |  | Lifetime any form of sexual   abuse | 18% |
|  |  | 1998: 548 AI/AN   adolescents | Lifetime sexual abuse by   family | 3% |
|  |  |  | Lifetime sexual abuse by   nonfamily member | 10% |
|  |  |  | Lifetime sexual abuse by   both family and nonfamily   members | 4% |
|  |  |  | Lifetime any form of sexual   abuse | 17% |
| Duran 2004 | Outpatient primary care clinic at community-based Indian Health Hospital in Albuquerque, clinic-based sampling with self-report survey | 234 AI/AN women  age 18-45 | At least one type of   maltreatment in childhood | 77% |
|  |  |  | Childhood neglect | 63% |
|  |  |  | Childhood emotional abuse | 55% |
|  |  |  | Childhood sexual abuse | 44% |
|  |  |  | Childhood physical abuse | 42% |
|  |  |  | Childhood sexual *and*   physical abuse | 28% |
| Libby 2005 | Southwestern tribe and 2 closely affiliated Northern Plains tribes; stratified random sampling of tribal rolls computer-assisted in-person interviews | *Southwest tribe*  1,446 Southwest tribal members age 15-54 | Childhood physical abuse   before age 13 in males | 7% |
|  |  |  | Childhood sexual abuse   before age 13 in males | 2% |
|  |  |  | Childhood physical abuse   before age 13 in females | 7% |
|  |  |  | Childhood sexual abuse   before age 13 in females | 8% |
|  |  |  | Childhood physical abuse   before age 13 in both   males and females | 7% |
|  |  |  | Childhood sexual abuse   before age 13 in both   males and females | 5% |
|  |  | *Northern Plains tribes*  1,638 Northern Plains tribal members age 15-54 (3,084 AI/AN participants total) | Childhood physical abuse   before age 13 in males | 7% |
|  |  |  | Childhood sexual abuse   before age 13 in males | 1% |
|  |  |  | Childhood physical abuse   before age 13 in females | 9% |
|  |  |  | Childhood sexual abuse   before age 13 in females | 7% |
|  |  |  | Childhood physical abuse   before age 13 in both   males and females | 8% |
|  |  |  | Childhood sexual abuse   before age 13 in both   males and females | 4% |
| Hanson 2006 | U.S. National household probability sample of 12-17 year olds with self-report survey | 139 AI/AN adolescents | Lifetime physically abusive punishment by caretakers | 15% |
|  |  |  | Lifetime intrafamilial sexual abuse | 4% |
|  |  |  | Lifetime extrafamilial sexual abuse | 11% |
| Saylors 2006 | Residential and outpatient substance abuse treatment settings in urban Oakland and San Francisco, California; self-report survey | 283 AI/AN women in treatment | Childhood sexual abuse | 56% |
|  |  |  | First incident at ages 1-5 | 37% |
|  |  |  | First incident at ages 6-10 | 37% |
| de Ravello 2008 | New Mexico prison system; self-report survey | 36 AI/AN incarcerated women age 20-60 years | Childhood sexually abuse | 53% |
|  |  |  | Childhood physically abuse | 42% |
|  |  |  | Childhood physically *and* sexually abuse | 33% |
|  |  |  | Childhood physical neglect | 22% |
| Hawkins 2010 | U.S. National household probability sample of 12-17 year olds with self-report survey | 86 AI/AN adolescents | Lifetime physical abuse by caretakers | 15% |
| Welch 2013 | Oklahoma Death Review Board files of fatalities attributed to child neglect; administrative data | 47 AI/AN child death cases | Fatality due to neglect | 13% |
|  |  |  |  |  |
|  | | | |  |
| Abbreviation: AI/AN, American Indian/Alaska Native | | | |  |
