# Supplementary material for: Family and partner interpersonal violence among American Indians/Alaska Natives
Source: Inj Epidemiol. 2014 Mar 20;1(1):7. doi: 10.1186/2197-1714-1-7 (PMC5005741; doi:10.1186/2197-1714-1-7)
Supplement: Supplementary file 2 — Authors’ original file for figure 2 [file 40621_2013_7_MOESM2_ESM.doc]

Table 2. Prevalence of violence against women among American Indians/Alaska Natives, by year of study

|  |  |  |  |  |  |
| --- | --- | --- | --- | --- | --- |
| First Author Year | Population and Data Source | Sample Size | Measure | Prevalence | |
| Curry 1998 | Prenatal clinics in a Northwest city; clinic-based sample with self-report survey | 57 AI/AN women | Past year physical IPV | 37% | |
|  |  |  | Past year sexual IPV | 7% | |
|  |  |  | IPV in pregnancy | 12% | |
| Fairchild 1998 | Indian Health Service  facility on a Navajo | 341 AI/AN women | Lifetime IPV | 53% | |
|  | Reservation; clinic-based sample with self-report survey |  | Lifetime physical IPV | 42% | |
|  |  |  | Lifetime sexual IPV | 12% | |
|  |  |  | Past year IPV | 16% | |
|  |  |  | Past year physical IPV | 14% | |
|  |  |  | Past year sexual IPV | 4% | |
| Kvinge 1998 | Pregnant women from a Northern Plains tribe; clinic-based sample with self-report survey | 177 AI/AN women | IPV in pregnancy | 30% | |
| Robin 1998 | Southwestern tribe; referral-based sampling with self-report survey | 56 married  AI/AN women | Lifetime IPV  Lifetime physical IPV  Lifetime sexual IPV  Past year IPV  IPV in pregnancy  IPV injuries requiring medical attention | 91%  79%  29%  31%  34%  46% | |
| Bohn 2002 | Prenatal clinic in Midwestern city; clinic-based sample with self-report survey | 30 AI/AN women | IPV in pregnancy  Threats to kill woman or her baby in pregnancy | 33%  6% | |
| Bohn 2003 | Prenatal clinic in a Midwestern city; clinic-based sample with self-report survey | 30 AI/AN women | Lifetime IPV | 87% | |
|  |  |  | Current partner IPV  Current partner physical IPV | 70%  60% | |
|  |  |  | Current partner sexual IPV | 17% | |
| Harwell 2003 | Seven reservations in Montana; random digit dial sample with self-report survey | 588 AI/AN women | Past year physical IPV | 3% | |
| Malcoe 2004 | WIC clinic in Oklahoma; clinic-based sample with self-report | 312 AI/AN women | Lifetime IPV  Lifetime sexual IPV  Lifetime threats with knife/gun  Past year IPV  Past year sexual IPV  IPV in pregnancy, past year  IPV resulting in injuries | 59%  12%  11%  30%  3%  9%  40% | |
| Simoni 2004 | New York City AI/AN community center members; self-report survey | 155 AI/AN women | Lifetime rape  Lifetime sexual IPV  Lifetime physical IPV | 34%  20%  31% | |
| Manson 2005 | 2 Northern Plains tribes and 1 Southwestern tribe; random sampling from tribal rolls with self-report survey | 829 AI/AN women in Southwest  848 AI/AN women in Northern Plains | Lifetime rape,  Southwest  Lifetime rape,  Northern Plains  Lifetime physical IPV,  Southwest  Lifetime physical IPV,  Northern Plains | 13%  14%  29%  31% | |
| Evans-Campbell 2006 | New York City AI/AN; respondent-driven, chain referral, and targeted sampling with self-report survey | 112 AI/AN women | Lifetime IPV | 40% | |
|  |  |  | Lifetime rape | 48% | |
| Yuan 2006 | Six tribes in the Southwest, Northwest, Northern Plains, and Northeast; random sample from tribal rolls with self-report survey | 744 AI/AN women | Lifetime rape | 14% | |
|  |  |  |  |  |  |
| Mylant 2008 | Teenage mothers in the Northern Plains participating in pregnancy program; self-report survey | 49 AI/AN women | Lifetime IPV  IPV in pregnancy  Past year sexual IPV | 61%  38%  23% | |
| Duran 2009 | Indian Health Services clinic in Albuquerque; clinic-based sample with self-report survey | 324 AI/AN women | Lifetime IPV | 80% | |
| Wood 2009 | Athabaskan tribe, Alaska; self-report survey | 91 AI/AN women | Lifetime IPV  Past year physical IPV  Lifetime threats with gun  Lifetime threats with knife | 64%  18%  19%  12% | |
| Rutman 2012 | National survey with self-report | 253 AI/AN women | Forced first sexual encounter | 17% | |
| Scott 2012 | Boarding school for at-risk AI/AN youth in a Plains state; self-report survey | 115 AI/AN women | Lifetime rape  Lifetime IPV | 20%  70% | |
| Ehlers 2013 | Eight contiguous reservations in California; respondent-driven and venue based sampling with self-report survey | 174 AI/AN women | Lifetime sexual abuse | 34% | |

Abbreviations: AI/AN, American Indian/Alaska Native; IPV, intimate partner violence
