# Supplementary material for: Family and partner interpersonal violence among American Indians/Alaska Natives
Source: Inj Epidemiol. 2014 Mar 20;1(1):7. doi: 10.1186/2197-1714-1-7 (PMC5005741; doi:10.1186/2197-1714-1-7)
Supplement: Supplementary file 3 — Authors’ original file for figure 3 [file 40621_2013_7_MOESM3_ESM.doc]

Table 3. Prevalence of elder abuse among American Indians/Alaska Natives, by year of study

| First Author Year | Population and Data Source | Sample size | Measure | Prevalence |
| --- | --- | --- | --- | --- |
| Brown 1989 | Navajo tribe, Oljato chapter; randomly selected tribal members with self-report survey. | Random sample of 37 AI/AN elderly from a population of 110 AI/AN elderly | Reported some extent of having been left alone and neglected when they needed help | 32% |
|  |  |  | Financially exploited by family members | 22% |
|  |  |  | Neglected in some way | 46% |
|  |  |  | Psychologically abused | 22% |
|  |  |  | Physically abused | 16% |
| Hudson 1998 | Two tribal groups in North Carolina; random sample of elderly tribal members with self-report survey. | 200 AI/AN;  92 over 65-years-old | Been abused at some time in life (all ages) | 26% |
|  |  |  | Abused after the age of 65 | 4% |
| Buchwald 2000 | Chart review of urban AI/AN in primary care in King County; clinic-based sample with medical chart review. | 550 AI/AN elderly age ≥50 | Definite or probable physical abuse | 10% |
|  |  |  | Suggestive physical abuse | 7% |
| Minton 2009 | 216 Alaska Natives from two rural Alaskan villages; randomly selected tribal members with structured interview responses. | 52 AI/AN elderly age ≥55 | Reported sadness as a result of victimization | 11% |

Abbreviation: AI/AN, American Indian/Alaska Native
