# Supplementary material for: Family and partner interpersonal violence among American Indians/Alaska Natives
Source: Inj Epidemiol. 2014 Mar 20;1(1):7. doi: 10.1186/2197-1714-1-7 (PMC5005741; doi:10.1186/2197-1714-1-7)
Supplement: Supplementary file 4 — Authors’ original file for figure 4 [file 40621_2013_7_MOESM4_ESM.doc]

Table 4. Risk factors for interpersonal violence in American Indian/Alaska Native children, women, and elders, by violence type

______________________________________________________________________________________________________________________________________________________

| First Author Year | Population | | Sample Size | | Risk Factor | | Outcome | |  |
| --- | --- | --- | --- | --- | --- | --- | --- | --- | --- |
| *Childhood Abuse* | | | | | | | | | |
| White 1981 | | Records Navajo children <9 years old | | 365 abused and/or neglected AI/AN children  867 comparison AI/AN children | | Unmarried parents | | Abuse or neglect | |
|  |  |  |  |  |  | Number of children in household | | Abuse or neglect | |
| Lujan 1989 | Southwestern Indian Health Service Hospital | | 117 medical records of abused and/or neglected AI/AN children ages 1-21 | | Alcohol abuse by caretaker | | Abuse or neglect | |  |
|  |  |  |  |  | History of abuse or neglect in caretaker | | Abuse or neglect | |  |
|  |  |  |  |  | Disability in child | | Abuse or neglect | |  |
| Nelson 1996 | Mesquakie tribe in Tama Country, Iowa; Siletz and other Northwest tribes in 11-county area in Northern Oregon | | 39 neglecting AI/AN families  38 comparison AI/AN families | | Mother under 19 years of age at first birth | | Neglect | |  |
|  |  |  |  |  | More than 1 father associated with household | | Neglect | |  |
|  |  |  |  |  | Number of children in household | | Neglect | |  |
|  |  |  |  |  | Parents separated or divorced | | Neglect | |  |
|  |  |  |  |  | Substance abuse in caretaker | | Neglect | |  |
|  |  |  |  |  | Criminal charges on caretaker’s record | | Neglect | |  |
|  |  |  |  |  | Caretaker has or is receiving psychiatric treatment | | Neglect | |  |
| *Violence Against Women* | | | | | | | | |  |
| Arbuckle 1996 | Female homicide victims in New Mexico | | 33 AI/AN women | | Alcohol or drug use by victim | | Homicide by partner | |  |
| Fairchild 1998 | Indian Health Service facility on a Navajo reservation | | 341 AI/AN women | | Receiving government assistance | | Current IPV | |  |
|  |  |  |  |  | Age (under 40) | | Current IPV | |  |
| Robin 1998 | Southwestern tribe | | 56 married AI/AN women | | Alcohol use by either perpetrator or victim | | Lifetime IPV | |  |
| Bohn 2003 | Clinic in a Midwestern city | | 30 AI/AN women | | Childhood abuse | | Adult abuse | |  |
| Harwell 2003 | Seven reservations in Montana | | 588 AI/AN women | | Age (under 45) | | Past year physical IPV | |  |
| Malcoe 2004 | WIC clinic in Oklahoma | | 312 AI/AN women | | Low SES (living at or below 50% poverty line; receiving government assistance; or partner with <HS education) | | Past year IPV | |  |
|  |  | |  | | Age (under 32) | | Past year IPV | |  |
|  |  | |  | |  | |  | |  |
|  |  | |  | | Separation/divorce | | Past year IPV | |  |
|  |  | |  | |  | |  | |  |
| Yuan 2006 | Six tribes in the Southwest, Northwest, Northern Plains, and Northeast | | 744 AI/AN women | | Alcohol dependence | | Physical assault | |  |
|  |  |  |  |  |  |  | Sexual assault | |  |
|  |  |  |  |  | Cohabitation | | Physical assault | |  |
|  |  |  |  |  |  |  | Sexual assault | |  |
|  |  |  |  |  | Separation/ divorce | | Physical assault | |  |
|  |  |  |  |  |  |  | Sexual assault | |  |
|  |  |  |  |  | Childhood sexual abuse | | Sexual assault | |  |
| *Elder Abuse* | | | | | | | | |  |
| Brown 1989 | One Navajo tribe, Oljato chapter (110 total elders) | | Random sample of 37 elderly | | Suddenness of onset of dependence on family | | Abuse overall | |  |
|  |  |  |  |  |  |  |  |  |  |
|  |  |  |  |  | Family perception of dependency | | Abuse overall | |  |
|  |  |  |  |  |  |  | Neglect | |  |
|  |  |  |  |  | Mental condition less than normal, as perceived by family | | Abuse overall | |  |
| Maxwell 1992 | 2 Plains Indians tribes | | Community-wide ethnographic study | | Caregivers who abused tended to be younger and live with their elders; abuse was more common among less wealthy tribe | | N/A | |  |
| Buchwald 2000 | Chart review of urban AI/AN (age >50) in primary care in King County | | 550 AI/AN elderly | | Younger age | | Definite or probable physical abuse | |  |
|  |  |  |  |  | Female | | Definite or probable physical abuse | |  |
|  |  |  |  |  | Currently depressed | | Definite or probable physical abuse | |  |
|  |  |  |  |  | More likely to depend on   others for food | | Definite or probable physical abuse | |  |

Abbreviations: AI/AN, American Indian/Alaska Native; IPV, intimate partner violence; OR, odds ratio
